# Descending Necrotizing Mediastinitis Resulting From Sialadenitis Without Sialolithiasis

**DOI:** 10.7759/cureus.24709

**Published:** 2022-05-03

**Authors:** Eric Silver, Nicholas Bial, Steve Yusupov

**Affiliations:** 1 Oral and Maxillofacial Surgery, Kings County Hospital Center, Brooklyn, USA; 2 Head and Neck Oncology, Staten Island University Hospital, Staten Island, USA

**Keywords:** infection, submandibular gland, descending necrotizing mediastinitis, mediastinitis, sialadenitis

## Abstract

Descending necrotizing mediastinitis (DNM) is an uncommon and life-threatening condition that arises from an oropharyngeal infection and descends along the cervical fascial planes into the mediastinum. Without aggressive surgical management, a high mortality rate exists. We report a case of an otherwise healthy 49-year-old male who presented with an abscess formation of the right submandibular gland secondary to sialadenitis without sialolithiasis. Computed tomography revealed fluid collection around the right submandibular gland suggestive of sialadenitis without sialolithiasis with severe inflammation and leftward deviation of the aerodigestive tract. Despite multiple drainages, the infection eventually progressed inferiorly into the mediastinum, resulting in DNM. After multiple takebacks to the operating room for exploration and washout of the neck and chest, intensive care unit management, and aggressive IV antibiotic therapy, the patient eventually had a successful recovery and was discharged home. In this paper, the etiology, anatomy, pathophysiology, and management of DNM are discussed. To our knowledge, this is the first report in the literature of DNM developing from sialadenitis without sialolithiasis in the submandibular gland.

## Introduction

Descending necrotizing mediastinitis (DNM) is a virulent polymicrobial infection that originates from an oropharyngeal source and descends into the mediastinum creating a potentially life-threatening condition. Delayed diagnosis and failure to appropriately recognize DNM can lead to rapid progression of sepsis and eventual death [1,2]. Mortality rates have been reported as between 20 and 50 percent and have generally not improved even in the era of antibiotics and 3D imaging [1,2]. DNM has been shown to statistically increase mortality rates in deep neck infections [3]. The criteria for diagnosing DNM include clinical evidence of severe pharyngeal infection, radiographic evidence of mediastinitis on CT scan, documenting the necrotizing mediastinal infection pre- and post-operatively, and an established relationship of the oropharyngeal infection and its development into DNM [4,5]. Risk factors include poor dentition, diabetes, AIDS, IV drug use, and excessive alcohol use. DNM presents more often in males than females [6-8]. Successful treatment consists of aggressive surgical drainage of the deep cervical spaces and mediastinum, broad-spectrum antimicrobial therapy, and upper airway management. The majority of DNM cases are odontogenic in origin, with less frequent causes including peritonsillar abscesses, retropharyngeal abscesses, cervical trauma, epiglottitis, and sialadenitis [2,9,10]. A glandular infection has been shown to be a higher risk factor for the development of DNM than an odontogenic infection [10]. Very few cases of DNM report submandibular sialadenitis with sialolithiasis. In this report, we discuss the successful treatment of DNM as a complication of sialadenitis without sialolithiasis. To our knowledge, this is the first reported case of DNM from a primary submandibular gland infection without sialolithiasis as a precipitating cause.

## Case presentation

A 49-year-old male with no significant past medical history or comorbidities presented to the emergency department with a chief complaint of right neck swelling and difficulty swallowing for four days. On physical examination, he was toxic appearing, restless, and unable to tolerate secretions with hoarseness and dysphonia. Trismus was minimal to none. Remarkable vitals signs were a temperature of 103.1° F, pulse rate of 109 beats per minute, respiratory rate of 22 breaths per minute, blood pressure of 137/87, and oxygen saturation of 97% on room air. White blood cell count was 18.66/mm^3^ with 83% neutrophils, and C-reactive protein was 239 mg/dL (Table 1).

**Table 1:** Patient's laboratory values on initial presentation Cells/mm^3^ - cells per cubic millimeter; mg/dL - milligrams per deciliter

Laboratory test	Value	Reference
White blood cell count	18.33 cells/mm^3^	4.5-11.5 cells/mm^3^
Neutrophils	83%	40-60%
C-reactive protein	239 mg/dL	<1.0 mg/dL

**Figure 1:**
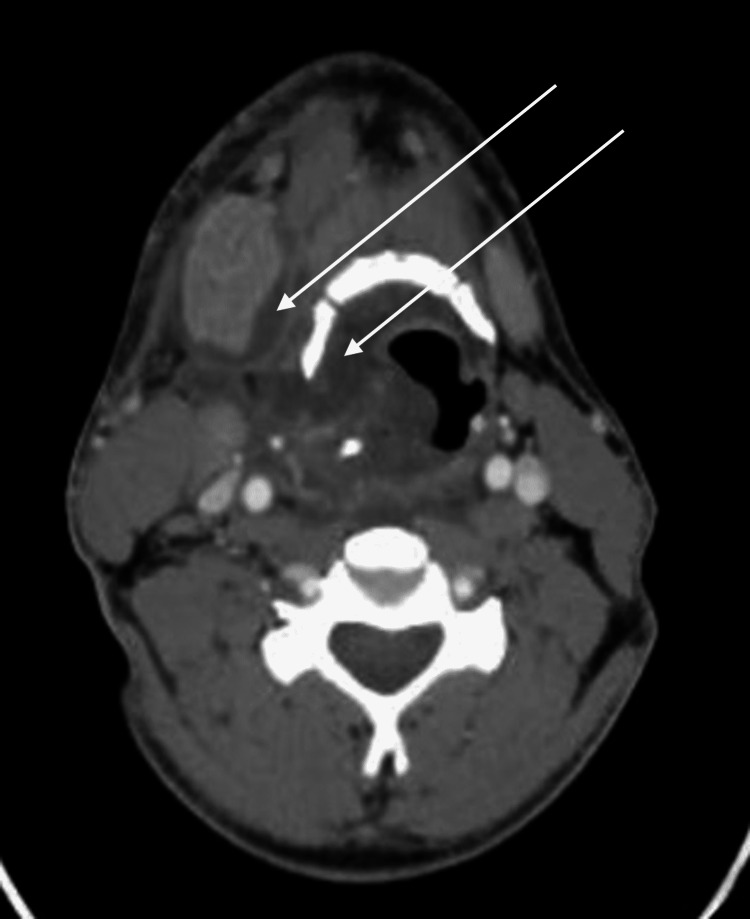
Axial view of CT scan at initial presentation There was significant enlargement and uniform enhancement of the right submandibular gland representing sialadenitis without sialolithiasis with severe surrounding inflammatory changes and mass effect upon the upper aerodigestive tract with severe deviation to the left. Multiloculated fluid (white arrows) was noted within the right aspect of the neck extending into the upper aerodigestive tract in several spaces, including the submandibular, submental, sublingual, paratracheal, and retropharyngeal spaces.

**Figure 2:**
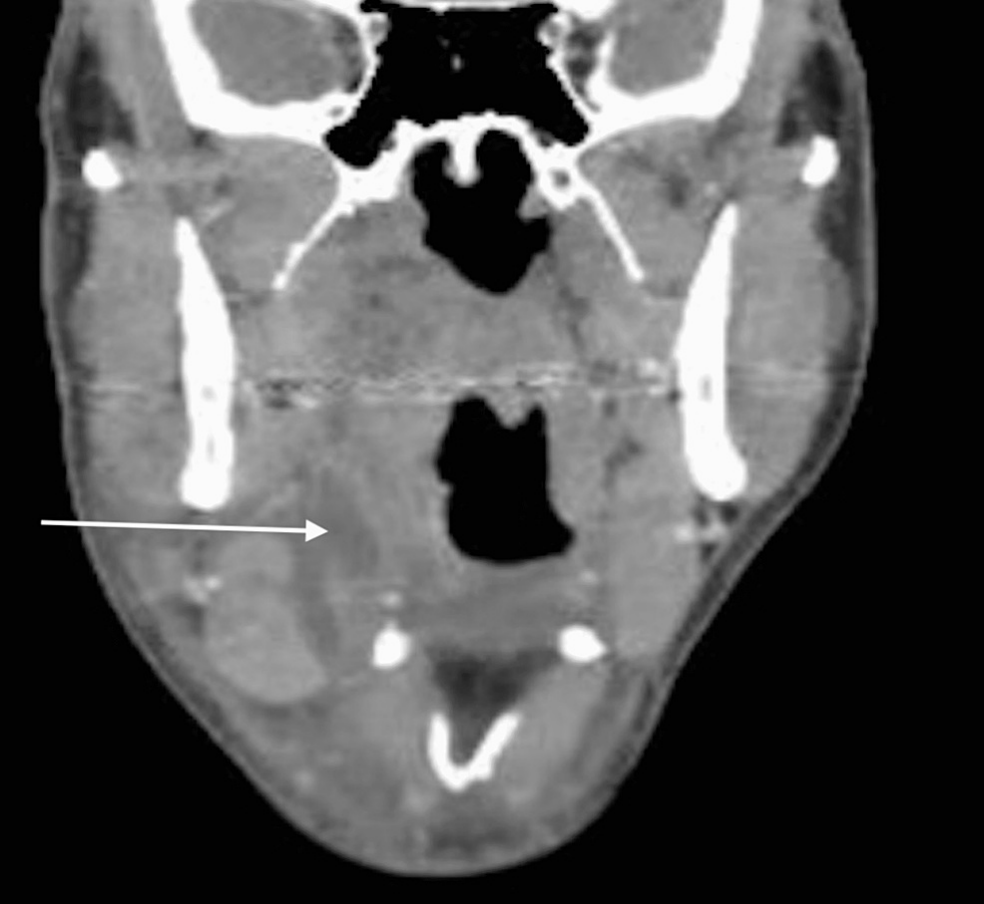
Coronal view of CT scan at initial presentation White arrows represent fluid collection surrounding the right submandibular gland.

COVID-19 and HIV results were negative. There was swelling of the right submandibular region with overlying erythema of the anterior neck extending inferiorly to the chest. The swelling was indurated and painful to palpation, particularly over the right submandibular gland. Oral examination revealed edema and erythema of the right tonsils and uvula. The teeth appeared healthy, and manipulation of the right submandibular gland failed to elicit any salivary drainage. Flexible laryngoscopy showed a sluggish and partially paralyzed right vocal cord with edema and partial obstruction of the glottis. Computed tomography (CT) scanning of the patient's head and neck showed significant enlargement and uniform enhancement of the right submandibular gland and mass effect of the aerodigestive tract deviating to the left. Unusual spaces, including paratracheal and parapharyngeal had rim enhancing collections, in addition to the submandibular, submental, and sublingual spaces (Figures 1,2). Findings were suggestive for acute suppurative sialadenitis without sialolithiasis.

The patient was emergently taken to the operating room for neck exploration and washout. Frank purulence (approximately 35 cc) was encountered in the right masticator, submandibular, and retropharyngeal spaces, as well as paratracheal and anterior neck compartments. The neck anatomic structures were found to be grossly inflamed and, due to gross purulence, were difficult to identify. A thorough washout with normal saline of the deep neck spaces was performed, and multiple gravity-dependent drains were placed in addition to iodoform packing. Iodoform packing changes were performed several times a day throughout the patient's hospital course. The gland excision was postponed at the initial drainage because the definitive source of infection had not been elucidated at the time, as well as to decrease the chances of facial, lingual, and hypoglossal nerve injury. Ultimately, the submandibular gland was not excised since the infection eventually spread well outside of the gland, involving other critical structures, then ultimately resolved. The patient remained intubated postoperatively and was transferred to the surgical intensive care unit for close airway monitoring. The patient initially showed improvement and was extubated on postoperative day 2. Initial wound cultures speciated Parvimonas and Prevotella. Ampicillin/sulbactam and vancomycin daily were initiated.

**Figure 3:**
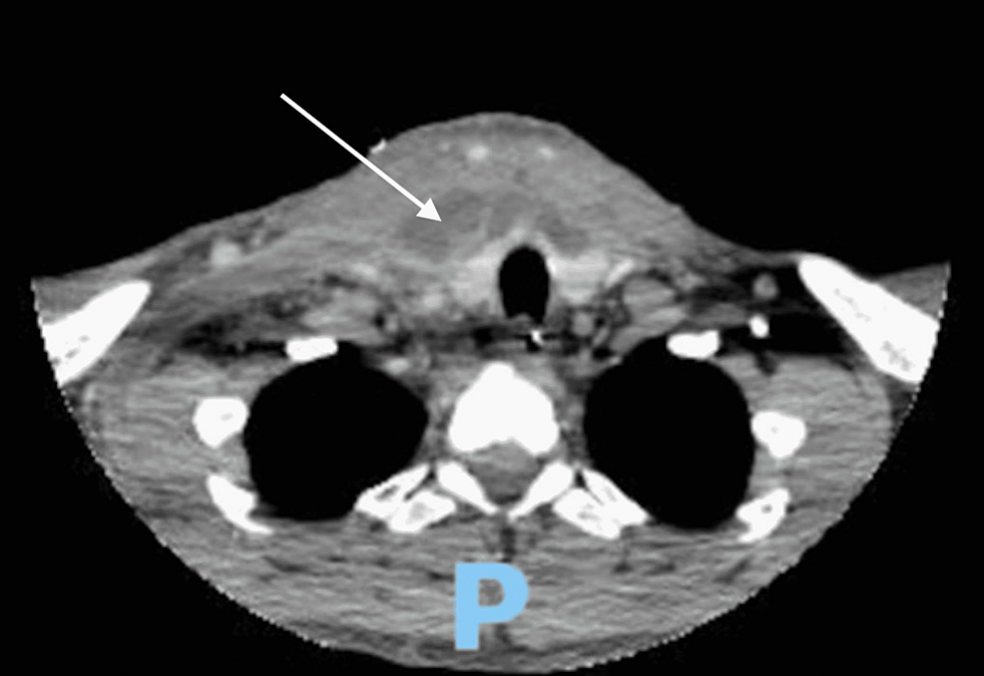
Axial view of CT scan on hospital day 5 Axial view of CT scan on hospital day 5 showed multiple fluid collections (white arrow) extending from the right submandibular space into the right carotid space, right prevertebral space, and the right retropharyngeal space. In addition, the chest CT showed communication of the right neck collections crossing midline and below the thyroid into the retropharyngeal space and inferiorly into the anterior and middle mediastinum compartments.

**Figure 4:**
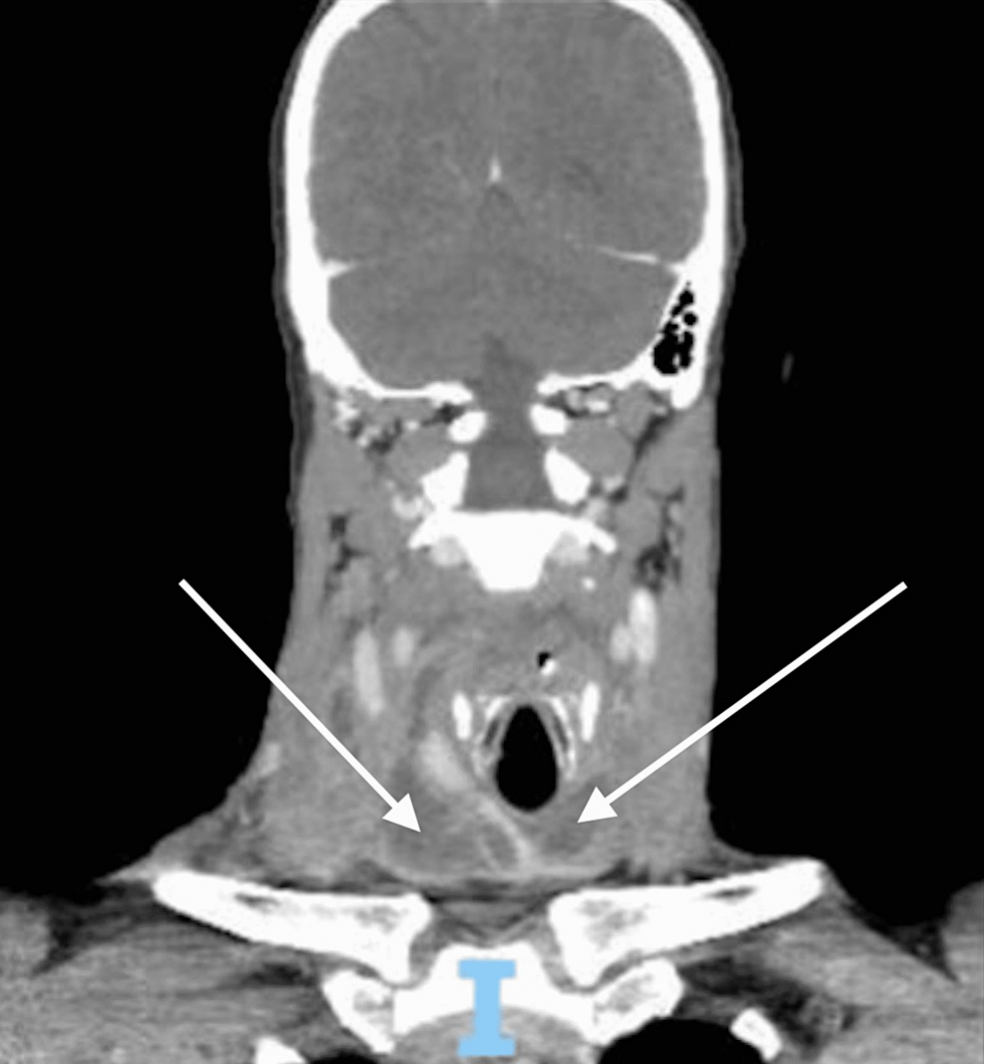
Coronal view of the CT scan on hospital day 5 White arrows represent fluid collection from descending infection.

**Figure 5:**
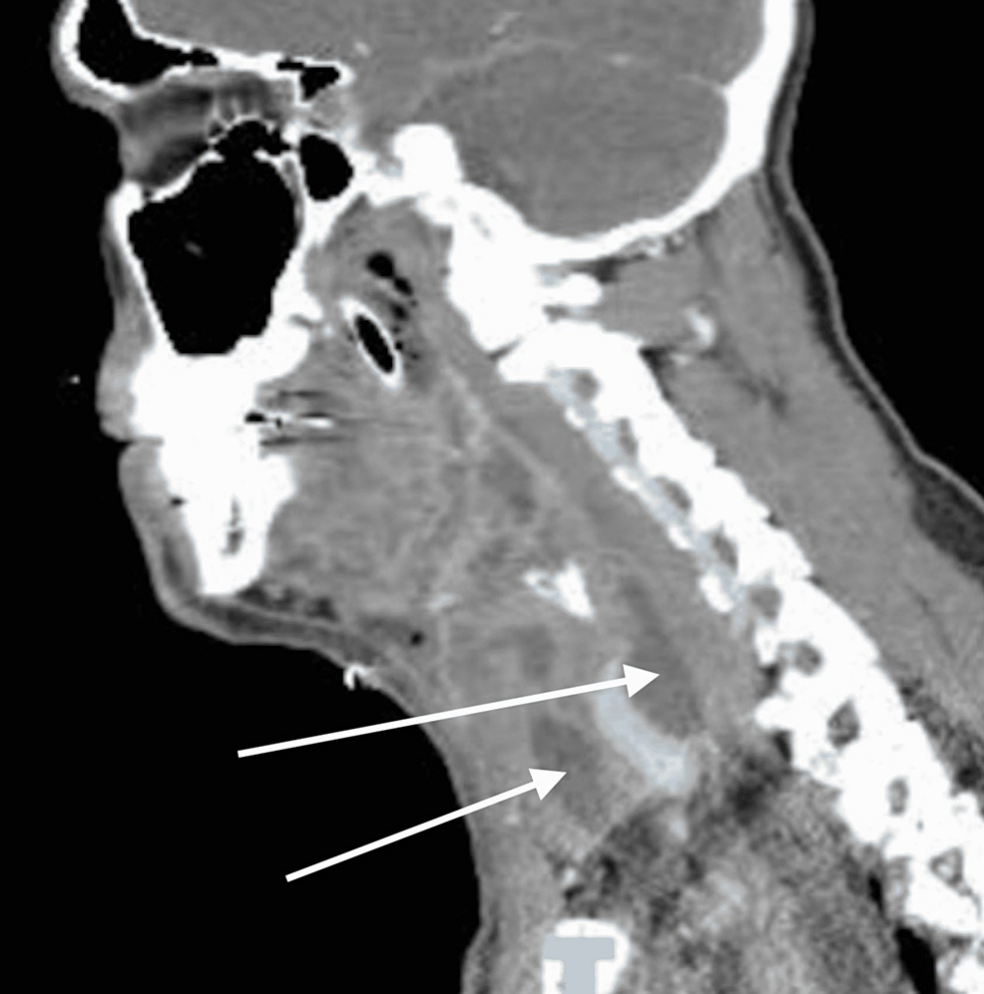
Sagittal view of the CT scan on hospital day 5 White arrows represent fluid collection from descending infection.

On hospital day 5, increased amounts of purulence were appreciated from the neck drains, and labs were remarkable for an up-trending white cell count (now 23.1/mm^3^). Repeat CT neck and chest showed multiple fluid collections extending from the right submandibular space into the right carotid space, right prevertebral space, and the right retropharyngeal space. Investigation of the chest CT showed communication of the right neck collections crossing midline and below the thyroid into the retropharyngeal space and inferiorly into the anterior and middle mediastinum compartments creating a 1.2 x 3.4 cm rim-enhancing collection (Figures 3-5). The patient was taken back to the operating room for re-exploration and washout of deep neck space infections, as well as mediastinal exploration and washout by thoracic surgery via right video-assisted thoracic surgery (VATS). Postoperatively chest and gastrostomy tubes were placed. Widespread necrosis of the superficial fascia in the submandibular, cervical, and mediastinal regions was encountered during the procedure. An extended neck exploration was performed intraoperatively, including dissection of the carotid sheath, retropharyngeal space, and the anterior neck compartment with dissection communicating with the mediastinum. Of note, minimal trismus was noted as the infection did not originate in the oral cavity. Therefore, intubations were performed with relative ease, and a tracheostomy did not need to be performed throughout the hospital course. The patient remained intubated in the cardiothoracic intensive care unit and was extubated on hospital day 7.

On hospital day 10, the patient again showed signs of clinical deterioration with a temperature of 101.2° F, white cell count of 32/mm^3^, complaints of unremitting chest pain, and purulence appreciated from his right superior pleural drain. A repeat CT neck and chest showed multiple small right supraclavicular loculations and a 5.2x7.0 cm right rim-enhancing infraclavicular collection involving the posterior mediastinum. Re-exploration and washout of the deep neck spaces and mediastinum were performed, and retropharyngeal and superior mediastinal drains were placed in direct communication to the pre-existing cervical drains. Antibiotics were transitioned to linezolid, piperacillin/tazobactam, and fluconazole. Significant clinical improvement was seen in the days to follow, and on hospital day 16, he was taken to the operating room for a final washout with the removal of neck packing. In total, the patient was taken to the operating room four times for drainage, washouts, and packing. Antibiotics were discontinued on hospital day 17. On hospital day 20, a peripherally inserted central catheter (PICC) line was placed, and all remaining drains and tubes were removed. On hospital day 22, he was discharged home. He received four weeks of outpatient IV ertapenem 1 g daily, and his recovery was satisfactory.

## Discussion

Descending necrotizing mediastinitis (DNM) is a major complication that can result from an uncontrolled episode of infection that originates in or travels to the deep cervical spaces. Fortunately, the disease process is rare, with approximately 100 cases having been reported in the literature [2]. Odontogenic infection is the most common source of DNM, occurring in 58% [1]. Other sources can include abscess of the retropharyngeal or peritonsillar spaces, abscess of the salivary or thyroid glands, lymphadenitis, traumatic intubation, or infection secondary to IV drug use [2]. Mortality rates remain high in this disease process despite advancements in diagnosis and care.

Typically, descending necrotizing mediastinitis originates from an odontogenic infection in the third molar region as a complication of Ludwig's angina. However, there are several other etiologies of DNM that can result from a primary deep neck infection, such as a retropharyngeal abscess, sialadenitis, or another deep cervical phlegmon/abscess [11]. Reported mortality rates of DNM are between 20% and 50%, even with prompt treatment, accurate diagnostic imaging, and appropriate antibiotics, making early recognition critical [11-13].

Certain deep cervical fascial spaces are interconnected to the mediastinum, providing a pathway for infection [1,2,11]. There are three layers of the deep cervical fascia; from superficial to deep they are the pretracheal, retrovisceral, and the prevertebral. These three layers create three potential spaces, named the pretracheal, perivascular, and retrovisceral (prevertebral) spaces, in which infection can descend inferiorly into the mediastinum. The pretracheal, perivascular, and retrovisceral spaces travel to the anterior, middle, and posterior mediastinum, respectively [1,11,14,15]. The retrovisceral space can be further divided into the retropharyngeal space and the danger (alar) space; these two entities are separated by the alar fascia. Rupture of the alar fascia provides access to the danger space, thus providing a pathway to the diaphragm and pleural spaces [11]. The carotid sheath is formed by the fusion of the three layers of the deep cervical fascia and surrounds the perivascular space, providing possible vascular complications [11,16,17]. Gravity and negative intrathoracic pressures contribute to mediastinal spread [2]. Approximately 70% of primary odontogenic infections that travel into the mediastinum do so via the retrovisceral space, and 8% do so via the pretracheal space [2,8].

Cultures of patients with DNM typically result in polymicrobial flora with aerobic and anaerobic bacteria [1,11,18]. In the case of our patient, initial wound cultures grew Parvimonas and Prevotella.

Management of DNM includes prompt diagnosis and urgent surgical intervention. Given the polymicrobial nature of DNM, broad-spectrum antibiotics are warranted. Surgical management of the neck should include source control as well as drainage, debridement, and washout. Intraoral and extraoral approaches may be used for adequate access. Surgical management and access to the mediastinum may include cervical drainage alone, but often more extensive intervention such as subxiphoid incision, open thoracotomy, VATS procedure, or transpleural drainage is necessary [1,2]. Access and extent of incision and drainage sites should be determined by the involved spaces on CT scan; however, clinicians should appreciate the rapidly evolving nature of the disease process and understand that the clinical extent of the disease may be more substantial than radiographic extent. Additionally, the threshold for operating room takeback should be low, and patients will often require multiple re-explorations in the operating room.

A classification system has been developed to describe different presentations of DNM. DNM type I has been described as located above the tracheal bifurcation, DNM type IIA has been described as extending into the anterior mediastinum below the tracheal bifurcation, and DNM type IIB has been described as extending into both the anterior and posterior mediastinum below the tracheal bifurcation [19]. These classifications may be used by cardiothoracic surgeons to guide the treatment of mediastinal involvement. Tracheostomy should be performed if there is any concern for airway compromise.

Despite advances in the understanding of the disease process and CT imaging, the mortality rate of DNM remains significant. Studies of DNM in the post-CT scan era have shown mortality rates of between 15% and 40%, which is similar to mortality rates in the pre-CT scan era (33%) [1,7]. Thus, early surgical intervention and open communication with other surgical teams are critical for adequate treatment outcomes of DNM.

In the case of our patient, the submandibular gland was found to be the primary source of infection. There have been reported cases of DNM with submandibular or parotid gland abscess as the primary source [6,10]. However, all these reported cases developed abscesses secondary to a salivary stone or did not explicitly state if a stone was involved. To the best of our knowledge, our patient is the first reported in the English language literature who developed DNM secondary to acute sialadenitis without sialolithiasis, which is a condition typically linked to salivary duct strictures, anticholinergic medication use, or predisposing factors such as diabetes mellitus, hypothyroidism, or Sjogren's syndrome [20]. This case is unique as the patient did not have any of the typical comorbidities associated with DNM, and it is unknown why the infection spread past the typical anatomical boundaries.

For the management of our patient, a combination of transcervical and transthoracic approaches was used for surgical access and drainage, and multiple takebacks to the operating room were required for adequate drainage. In addition, broad-spectrum antibiotics and multiple daily packing with dressing changes were required. The patient did not require a tracheostomy as his airway remained patent throughout his hospital course.

## Conclusions

Descending necrotizing mediastinitis (DNM) is a rare but feared complication of a primary deep neck infection. Due to anatomical connections between spaces in the deep neck and mediastinum, the infection can travel freely, requiring extensive drainage. Treatment includes prompt airway management, source control with aggressive surgical intervention, and tailored broad-spectrum antibiotics. We present the first case in the literature of sialadenitis without sialolithiasis that resulted in DNM. The patient was successfully treated with aggressive exploration and washouts of the cervical and superior mediastinal compartments, intensive care management with careful airway monitoring, and continuous antibiotic therapy. Despite early recognition, the patient required a lengthy hospital stay and multiple takebacks to the operating room.
